# Identifying gut microbiota with high specificity for ischemic stroke with large vessel occlusion

**DOI:** 10.1038/s41598-024-64819-6

**Published:** 2024-06-18

**Authors:** Ping He, Chen Jiang, Jianqiang Ni, Xiaoxuan Zhang, Zhifeng Wu, Gengjing Chen, Jin Huang, Zheng Dai, Wei Ji, Lei Li, Kefei Chen, Yachen Shi

**Affiliations:** 1 Department of Neurosurgery Intensive Care Unit, The Affiliated Wuxi People’s Hospital of Nanjing Medical University, Wuxi People’s Hospital, Wuxi Medical Center, Nanjing Medical University, Wuxi, 214023 China; 2 Department of Neurology, The Affiliated Wuxi People’s Hospital of Nanjing Medical University, Wuxi People’s Hospital, Wuxi Medical Center, Nanjing Medical University, Qingyang Road No. 299, Wuxi, 214023 Jiangsu China; 3 Department of Neurology, The First Affiliated Hospital of Soochow University, Suzhou, 215000 China; 4 Department of Neurosurgery, The Affiliated Wuxi People’s Hospital of Nanjing Medical University, Wuxi People’s Hospital, Wuxi Medical Center, Nanjing Medical University, Wuxi, 214023 China; 5 Department of Interventional Neurology, The Affiliated Wuxi People’s Hospital of Nanjing Medical University, Wuxi People’s Hospital, Wuxi Medical Center, Nanjing Medical University, Wuxi, 214023 China; 6 Department of Functional Neurology, The Affiliated Wuxi People’s Hospital of Nanjing Medical University, Wuxi People’s Hospital, Wuxi Medical Center, Nanjing Medical University, Qingyang Road No. 299, Wuxi, 214023 Jiangsu China

**Keywords:** Microbiology, Diseases, Neurology, Risk factors

## Abstract

Gut microbiota can regulate the metabolic and immunological aspects of ischemic stroke and modulate the treatment effects. The present study aimed to identify specific changes in gut microbiota in patients with large vessel occlusion (LVO) ischemic stroke and assess the potential association between gut microbiota and clinical features of ischemic stroke. A total of 63 CSVD patients, 64 cerebral small vessel disease (CSVD) patients, and 36 matching normal controls (NCs) were included in this study. The fecal samples were collected for all participants and analyzed for gut microbiota using 16S rRNA gene sequencing technology. The abundances of five gut microbiota, including genera Bifidobacterium, Butyricimonas, Blautia, and Dorea and species Bifidobacterium_longum, showed significant changes with high specificity in the LVO patients as compared to the NCs and CSVD patients. In LVO patients, the genera Bifidobacterium and Blautia and species Bifidobacterium_longum were significantly correlated with the National Institutes of Health Stroke Scale (NIHSS) scores at the admission and discharge of the patients. Serum triglyceride levels could significantly affect the association of the abundance of genus Bifidobacterium and species Bifidobacterium_longum with the NIHSS scores at admission and modified Rankin Scale (mRS) at discharge in LVO patients. The identification of five gut microbiota with high specificity were identified in the early stage of LVO stroke, which contributed to performed an effective clinical management for LVO ischemic stroke.

## Introduction

Acute stroke is a life-threatening disease and a major cause of serious disability^1^. Large vessel occlusion (LVO) is typically caused by a thrombus or embolus that lodges into one of the large vessels of the intracranial circulation, diminishing cerebral blood flow to cause brain cell death and acute ischemic stroke^2,3^. Several scales [e.g., National Institutes of Health Stroke Scale (NIHSS)] have been considered to predict the occurrence of LVO stroke^4^; however, no proper means have proven sensitive and feasible enough yet. Meanwhile, although large artery atherosclerosis and cardioembolism are the two main reasons, the variable and complex pathogenesis can also cause LVO, such as cerebral vasculitis, dissecting aneurysm^5,6^. Furthermore, endovascular treatment and intravenous thrombolysis are the common treatments for acute LVO^7,8^; however, the subsequent drug therapy and clinical prognosis have not been sufficiently investigated. Therefore, it is essential to study the underlying pathological mechanisms and predictors of clinical outcomes in stroke patients with LVO.

The gut microbiota is the largest reservoir of microorganisms in the human body and plays an important role in regulating the metabolic and immunological aspects of ischemic stroke^9,10^. Previous studies have demonstrated an altered composition and abundance of the gut microbiota in various complications and poor outcomes post-stroke, including *Streptococcus*, *Lactobacillus,* and *Oscillospira*^11,12^. Additionally, the dysregulation of gut microbiota, by causing abnormal metabolism of short-chain fatty acids, can mediate immune and inflammatory reactions within the central nervous system and further affect stroke outcomes^13,14^. However, the investigation into the role of the gut-brain axis in a stroke is in its initial stage, and the association of the gut microbiota with the development and clinical outcome of an ischemic stroke needs to be clarified.

The present study aimed to identify specific gut microbiota in LVO stroke, which differed from the cerebral small vessel disease (CSVD) and normal state. Meanwhile, the association between gut microbiota and LVO stroke, including clinical symptoms, blood indices levels, and therapeutic effect, was also assessed.

## Results

### Characteristics of participants

Table 1 represents no difference in age, sex, body mass index (BMI), and complications (hypertension, diabetes, and coronary heart disease) among normal control (NC), CSVD and LVO groups.

**Table 1:** Clinical characteristic of participants in the present study.

	NC (n = 36)	CSVD (n = 64)	LVO (n = 63)	p-value
Age (years)	66.75 ± 5.86	69.16 ± 5.73	66.25 ± 10.96	0.114*
Sex (Male, %)	20 (55.55%)	32 (50.00%)	43 (68.25%)	0.106^#^
BMI	24.04 ± 2.97	23.73 ± 2.33	24.67 ± 2.53	0.110*
Complications (N, %)				
Hypertension	15 (41.67%)	38 (59.38%)	42 (66.67%)	0.051^#^
Diabetes	6 (16.67)	17 (26.56%)	23 (36.51%)	0.100^#^
Coronary heart disease	1 (2.78%)	7 (10.93%)	3 (4.76%)	0.214^#^
Admission NIHSS scores	–	–	7.58 ± 6.01	–
Discharge NIHSS scores	–	–	4.81 ± 6.26	–
Admission mRS scores	–	–	3.15 ± 1.33	–
Discharge mRS scores	–	–	1.67 ± 1.85	–
BIV (cm^3^)	–	–	20.50 ± 54.00	–
Stroke blood risk indices				
TC (mmol/L)	–	–	4.65 ± 1.31	–
TG (mmol/L)	–	–	2.08 ± 1.69	–
LDL-C (mmol/L)	–	–	2.60 ± 0.89	–
HDL-C (mmol/L)	–	–	1.16 ± 0.52	–
FBG (mmol/L)	–	–	6.79 ± 2.46	–
HbAlc (%)	–	–	6.80 ± 1.59	–
HCY (μmol/L)	–	–	15.16 ± 5.49	–
UA (μmol/L)	–	–	289.05 ± 109.64	–

Data are presented as the mean ± stand deviation or number of participants in each group (% of total).

NC, normal control; CSVD, cerebral small vessel disease; LVO, large vessel occlusion, BMI, body mass index; NIHSS, National Institutes of Health Stroke Scale; mRS, modified Rankin Scale; BIV, brain infarct volume; TC, total cholesterol; TG, triglyceride; LDL-C, low-density lipoprotein cholesterol; HDL-C, high-density lipoprotein cholesterol; FBG, fasting blood-glucose; HbAlc, hemoglobin A1c; HCY, homocysteine; UA, uric acid.

*p values were obtained by One-way ANOVA test.

^&^p values were obtained by Kruskal–Wallis H test.

^#^p values were obtained by Chi-square test.

### Compositional analysis of gut microbiota in CSVD and HC groups

A total 8,439,651 sequences were obtained from 165 samples using QIIME2 software (version 2023.02; URL link: https://qiime2.org/), including 1,863,972 sequences in the NC group, 3,313,728 sequences in the CSVD group, and 3,261,951 sequences in the LVO group.

The operational taxonomic units (OTUs) were assigned with a 95% sequence similarity threshold. The LVO group exhibited a higher number of OTUs than the NC and CSVD groups (5028 vs. 2626 vs. 3455), including 956 similar OTUs (Supplementary Fig. S1). Samples’ curves in the rarefaction curves based on the amplicon sequence variant reached saturation plateau at the depth of 51,777 reads, signifying that the sequencing depths were sufficient for the majority of microbe species, and the sample size was reasonable (Supplementary Fig. S2).

### Diversity analysis of gut microbiota among three groups

#### Alpha diversity analysis

**Figure 1:**
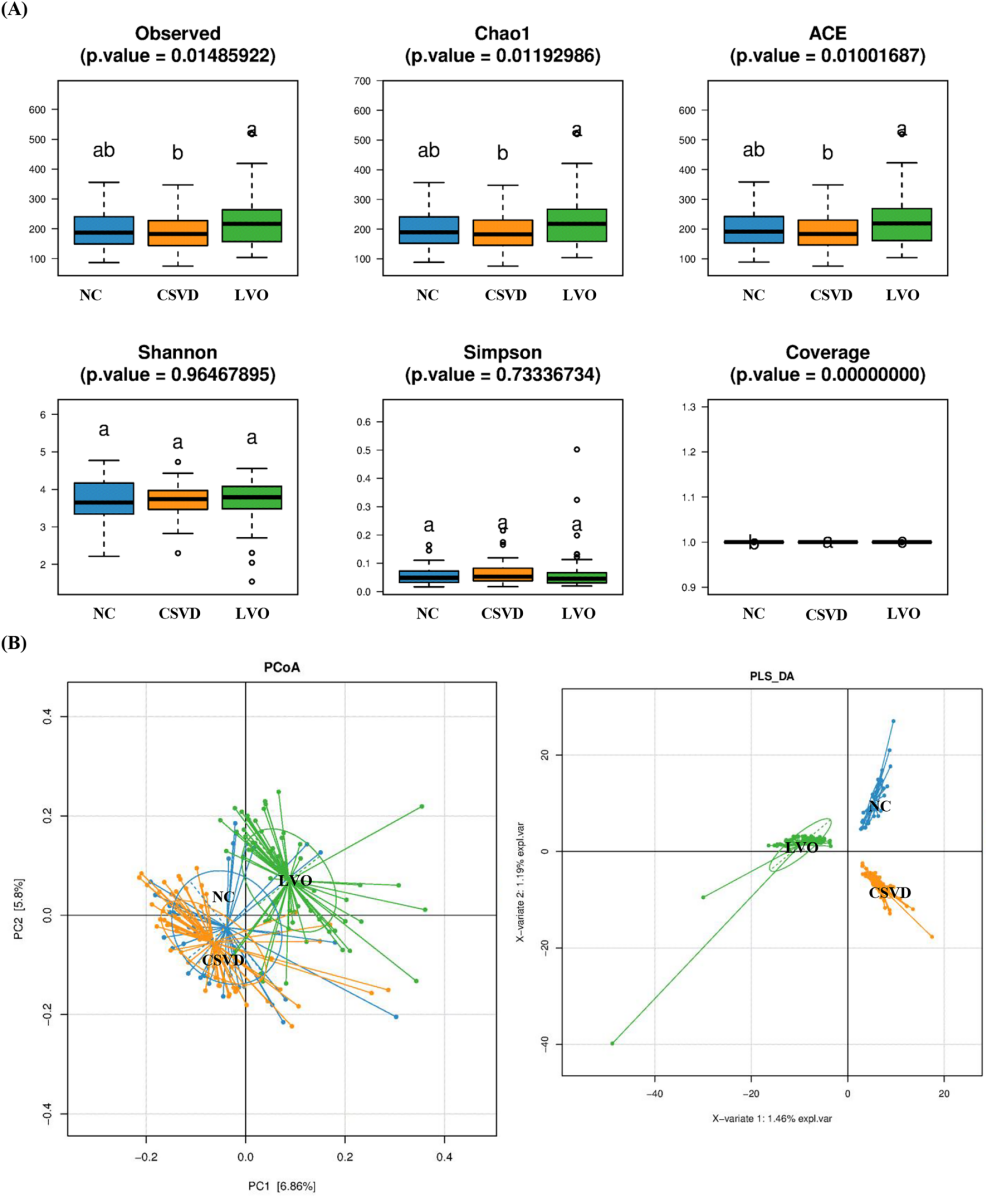
Alpha-diversity and Beta-diversity indices for the species in the gut microbiota among NC, CSVD and LVO groups. (**A**) α-diversity indices. Same letter (a or b) on the box indicates no significant difference in the index between two groups. (**B**) β-diversity indices. Principal coordinates (PCoA) analysis (Left). Axis. 1 and 2 are two main components with the most interpretation of differences between samples. PLS-DA analysis (Right). The scale stands for the relative distance without practical significance. The NC, CSVD and LVO subjects are colored in blue, orange and green, respectively. NC, normal control; CSVD, cerebral small vessel disease; LVO, large vessel occlusion.

Six α-diversity indices, i.e., Observed species, Chao1, ACE, Shannon, Simpson and Coverage, were included in the present study (Fig. 1A). The scores of Observed species, Chao1, and ACE showed significant difference among NC, CSVD and LVO groups (F_Observed species_ = 4.322; F_Chao1_ = 4.553; F_ACE_ = 4.739; F_Coverage_ = 29.301). Using the post-hoc analysis, LVO patients had significant higher Observed species, Chao1, and ACE scores than CSVD patients. Meanwhile, among NC, CSVD, and LVO groups, Coverage scores showed significantly difference (F_Coverage_ = 29.301), however, there were no significant differences found in Shannon and Simpson scores.

#### Beta diversity analysis

The β-diversity was calculated by Principal Coordinate Analysis (PCoA) analysis based on the weighted UniFrac distances at OTU level (Fig. 1B). Meanwhile, the Partial Least Squares-Discriminant Analysis (PLS-DA) analysis was also conducted for the reduction of the impact of intergroup differences (Fig. 1B).

#### Compositional analysis of gut microbiota at genus and species level levels among three groups

The top one most dominant bacterial genera with the highest relative abundances in these three groups were consistent, i.e., *Bacteroidetes* (Supplementary Fig. S3). *Prevotella*, as the second highest abundant bacterium, only appeared in the LVO group, however, the second highest abundance bacterium of the NC and CSVD groups was *Faecalibacterium* (Supplementary Fig. S3). Meanwhile, in species level, *Bacteroides_vulgatus* as the recognizable bacteria showed the consistently highest relative abundances in three groups (Supplementary Fig. S4). *Bacteroides_uniformis* was the second highest abundance bacterium in both the NC and CSVD groups, but *Bacteroides_coprocola* was the second highest abundance bacterium in the LVO group (Supplementary Fig. S4).

**Figure 2:**
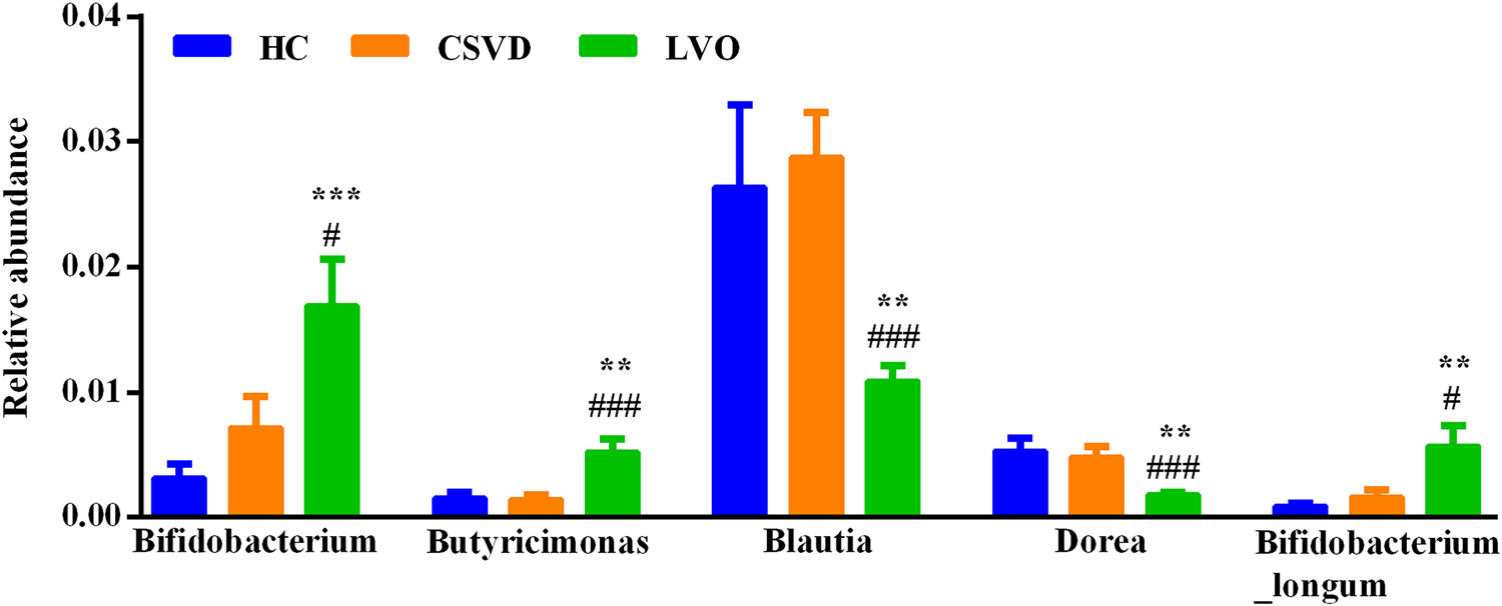
Taxa with significant differences at phylum and species level among NC, CSVD and LVO groups. “*” and “^#^” indicate significant changes in the LVO group as compared to the NC or CSVD group, respectively. ***^/###^p-value < 0.001; **^/##^p-value < 0.01; *^/#^p-value < 0.05. NC, normal control; CSVD, cerebral small vessel disease; LVO, large vessel occlusion.

There were 20 genera and 17 species, having significant differences in their relative abundances, among the NC, CSVD and LVO groups (Supplementary Figs. S5 and S6). Using the MetaStats analysis, four genera and one species with specifically changed relative abundances in the LVO group (Fig. 2). In genus level, relative abundances of *Bifidobacterium* and *Butyricimonas* significantly increased and relative abundances of *Blautia* and *Dorea* significantly reduced in the LVO group when compared to HC and CSVD groups (Fig. 2). Moreover, LVO patients also exhibited significantly higher relative abundances in *Bifidobacterium_longum* than HC and CSVD participants in species level (Fig. 2). Therefore, these five gut microbes showed significant changes with high specificity in LVO patients. Moreover, LVO patients were further divided into two subgroups, *i.e.,* cardioembolism and large-artery atherosclerosis groups (Supplementary Table 1). The relative abundances of these five gut microbes showed no significant difference between cardioembolism and large-artery atherosclerosis subgroups (Supplementary Fig. S7). Furthermore, gender as an important factor was also considered for the expression of gut microbes. There was no significant difference in the relative abundances of these five gut microbes between male and female subgroups in LVO group (Supplementary Fig. S8).

#### Results of linear discriminant analysis effect size (LEfSe) analysis

Using the LEfSe analysis, there were 91 taxa been identified with Linear Discriminant Analysis (LDA) scores of > 2 and p-value of < 0.05. Supplementary Fig. S9A showed a cladogram for all the taxonomic levels abundance, and the top 10 taxa with the highest LDA scores in each group are shown in Supplementary Fig. S9B. As shown in Supplementary Fig. S9, at genus level, the LVO group had significantly changed relative abundance of *Actinomyces, Bifidobacterium, Scardovia, Atopobium*, *Cryptobacterium*, *Bacteroides*, *Parabacteroides*, *Alloprevotella*, *Prevotella*, *Enterococcus*, *Lactobacillus*, *Ezakiella*, *Hungatella*, *Anaerotruncus*, *Megasphaera*, *Fusobacterium*, *Campylobacter,* and *Succinivibrio* as compared to the HC and CSVD groups. Meanwhile, compared with the NC and CSVD groups, species *Clostridium_symbiosum* and *Sutterella_wadsworthensis* also showed significant difference in relative abundance in the LVO group (Supplementary Fig. S9).

#### Functional predictions for gut microbiota

**Figure 3:**
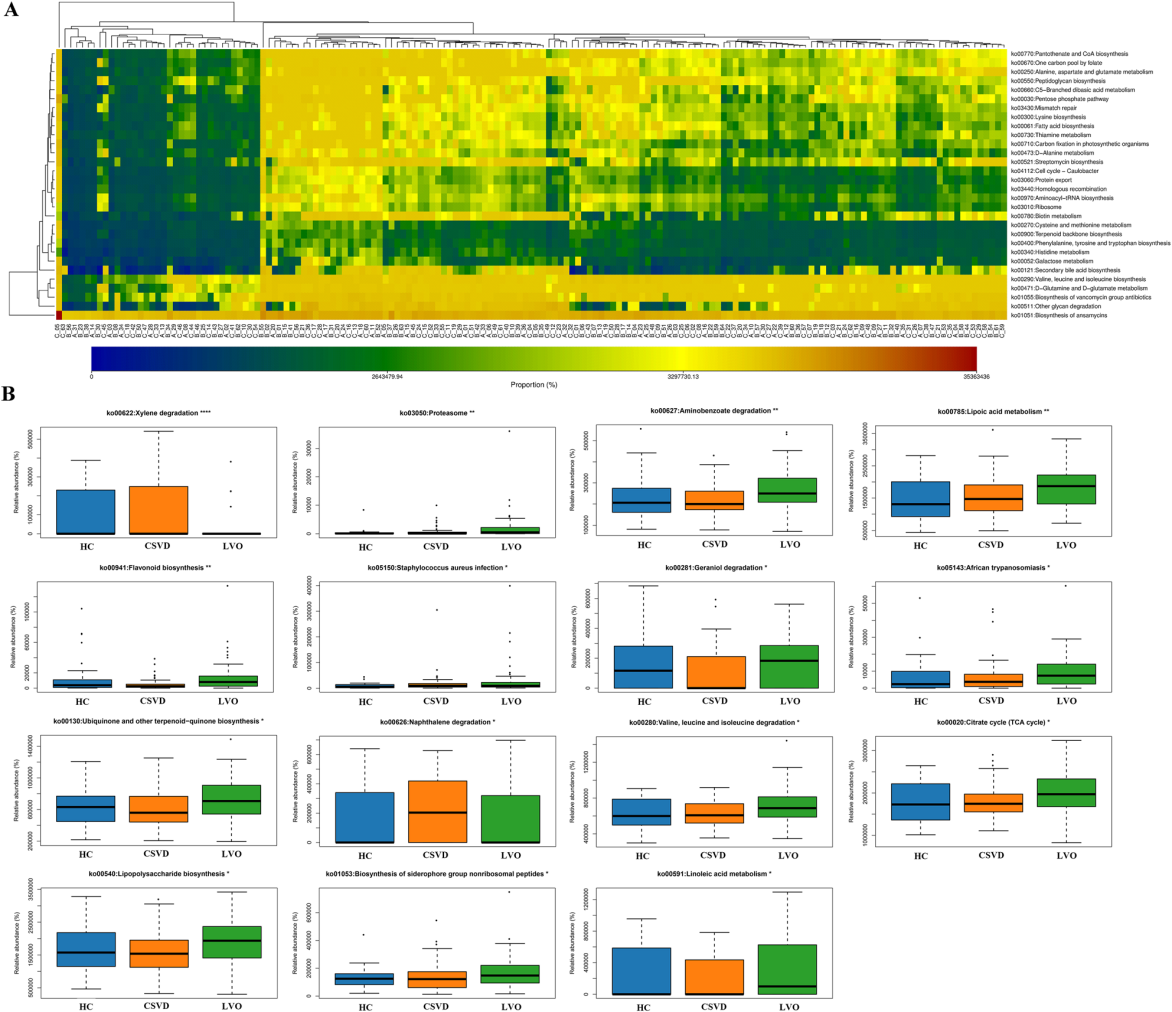
Functional predictions of the gut microbiota. (**A**) Heat-map of functional genes in gut microbiota of all participants, showing top 30 genes with maximum relative abundances. The Abscissa stands for the samples, and the ordinate is functional genes. The colors represent the abundance of function, and the gradual change in color from light to deep indicates the relative abundance of function from low to high. (**B**) Significant difference in Kyoto Encyclopedia of Genes and Genomes pathways for gut microbiota in three groups using Kruskal–Wallis test. ***p-value < 0.001; **p-value < 0.01; *p-value < 0.05. The red boxes indicate significant changes in the LVO group as compared to the NC and CSVD group. NC, normal control; CSVD, cerebral small vessel disease; LVO, large vessel occlusion.

Using the PICRUSt2 analysis tool, the underlying functions in the gut microbiota of the three groups were predicted and annotated based on the Kyoto Encyclopedia of Genes and Genomes database (Fig. 3A). The differences in the functional prediction among the three groups were further analyzed using Kruskal–Wallis test. The pathway of “Xylene degradation”, “Proteasome”, “Aminobenzoate degradation”, “Lipoic acid metabolism”, “Flavonoid biosynthesis”, “Staphylococcus aureus infection”, “Geraniol degradation”, “African trypanosomiasis”, “Ubiquinone and other terpenoid-quinone biosynthesis”, “Naphthalene degradation”, “Valine, leucine and isoleucine degradation”, “Citrate cycle (TCA cycle)”, “Lipopolysaccharide biosynthesis”, “Biosynthesis of siderophore group nonribosomal peptides” and “Linoleic acid metabolism” showed significant difference among three groups (Fig. 3B). Additionally, using Wilcoxon test, the relative abundances of “Xylene degradation” were significantly reduced and the relative abundances of “Proteasome”, “Lipoic acid metabolism”, “Aminobenzoate degradation”, “African trypanosomiasis”, and “Citrate cycle (TCA cycle)” were significantly increased in the LVO group as compared to the NC or CSVD group (Fig. 3B).

### Association analysis of environmental factors in LVO patients

#### Correlation analysis

**Figure 4:**
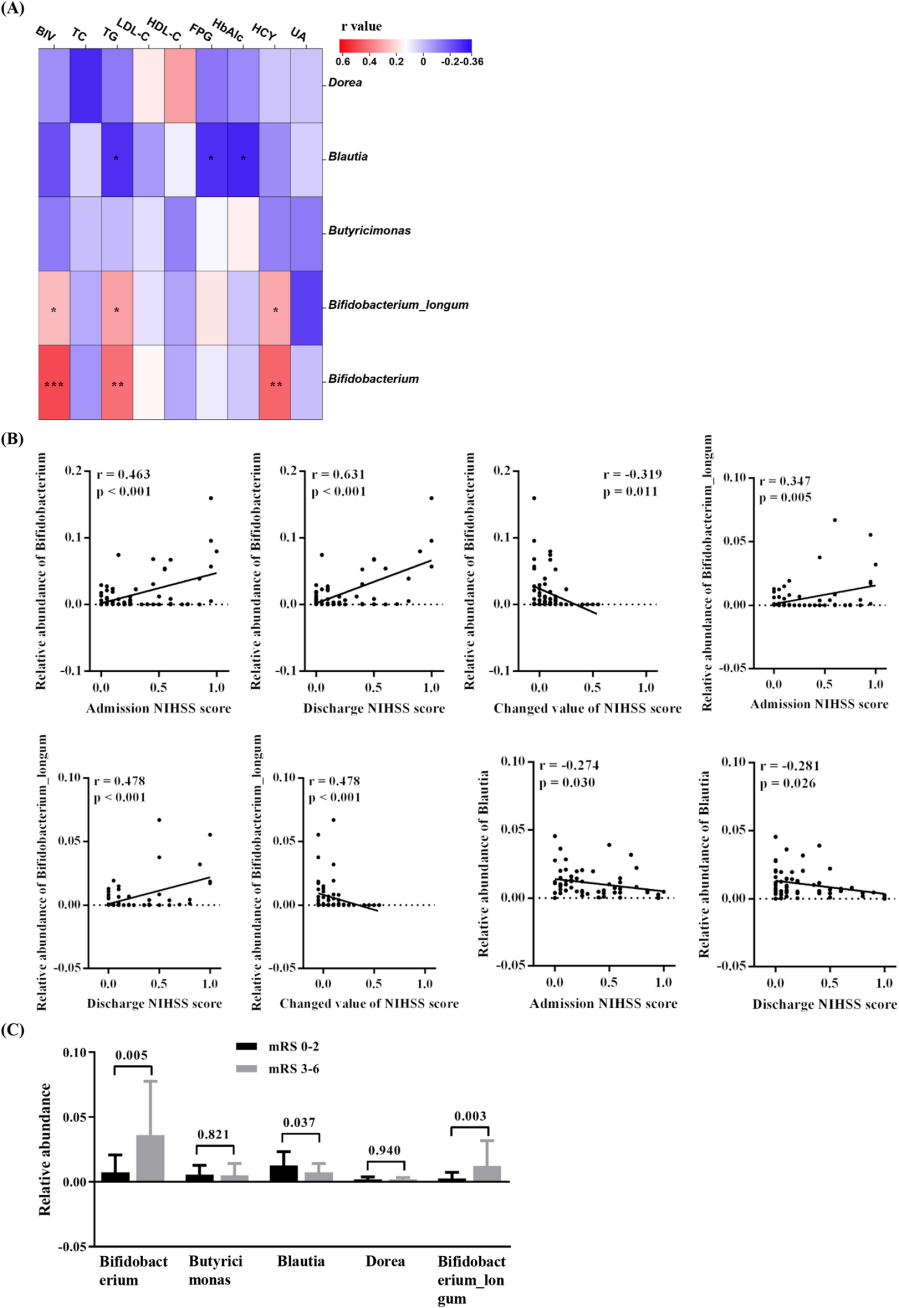
Associations of clinical features with gut microbiota at genus and species level in LVO patients. (**A**) Heatmap shows the correlation coefficients between clinical features at admission and gut microbiota. Covariates were age, sex, BMI, and complications. (**B**) Correlation analyses of NIHSS scores at admission and discharge with gut microbiota. Original NIHSS score was normalized to [0, 1] using Min–Max Normalization. Changed value = admission score − discharge score. Covariates were age, sex, BMI, and complications. (**C**) Difference in gut microbiota at genus and species level between good functional outcome and poor functional outcome groups. Good functional outcome was defined as a mRS score of 0–2, and poor functional outcome was defined as a mRS score of 3–6. LVO, large vessel occlusion, NIHSS, National Institutes of Health Stroke Scale; mRS, modified Rankin Scale; BIV, brain infarct volume; TC, total cholesterol; TG, triglyceride; LDL-C, low-density lipoprotein cholesterol; HDL-C, high-density lipoprotein cholesterol; FBG, fasting blood-glucose; HbAlc, hemoglobin A1c; HCY, homocysteine; UA, Uric Acid; BMI, body mass index.

In order to determine the potential correlations between the composition of gut microbiota and environmental factors in LVO patients, one correlation matrixes were generated using partial correlation analysis with adjusting age, sex, BMI, and complications (Fig. 4A). Five gut microbes that showed specific changes in patients with LVO were included in the present analysis. In LVO patients, the relative abundance of genera *Bifidobacterium* and species *Bifidobacterium_longum* showed significant positive correlations with brain infarction volume (BIV) and triglyceride (TG), and homocysteine (HCY) levels. Meanwhile, LVO patients had significant negative correlations between the TG, fasting blood-glucose (FBG), and hemoglobin A1c (HbAlc) levels.

To better analyze the data, the original NIHSS score was normalized to [0,1] using Min–Max Normalization. The relative abundance of genera *Bifidobacterium* and species *Bifidobacterium_longum* showed significant positive correlations with admission and discharge NIHSS scores and negatively correlated with changed value of NIHSS (Fig. 4B). Meanwhile, there were significant negative correlation between the relative abundance of genera *Blautia* and admission and discharge NIHSS scores in the LVO group (Fig. 4B).

Furthermore, LVO patients were further divided into the good functional outcome group (score of 0–2) and the poor functional outcome group (score of 3–6) according to the discharge modified Rankin Scale (mRS) scores^15,16^. No significant difference in age, sex, BMI, and complications (hypertension, diabetes and coronary heart disease) between these two groups (data not shown). The relative abundance of genera *Bifidobacterium* and species *Bifidobacterium_longum* were significantly increased and the relative abundance of genera *Blautia* were significantly reduced in the poor functional outcome group as compared to the good functional outcome group in LVO patients (Fig. 4C).

#### Mediation analysis

**Figure 5:**
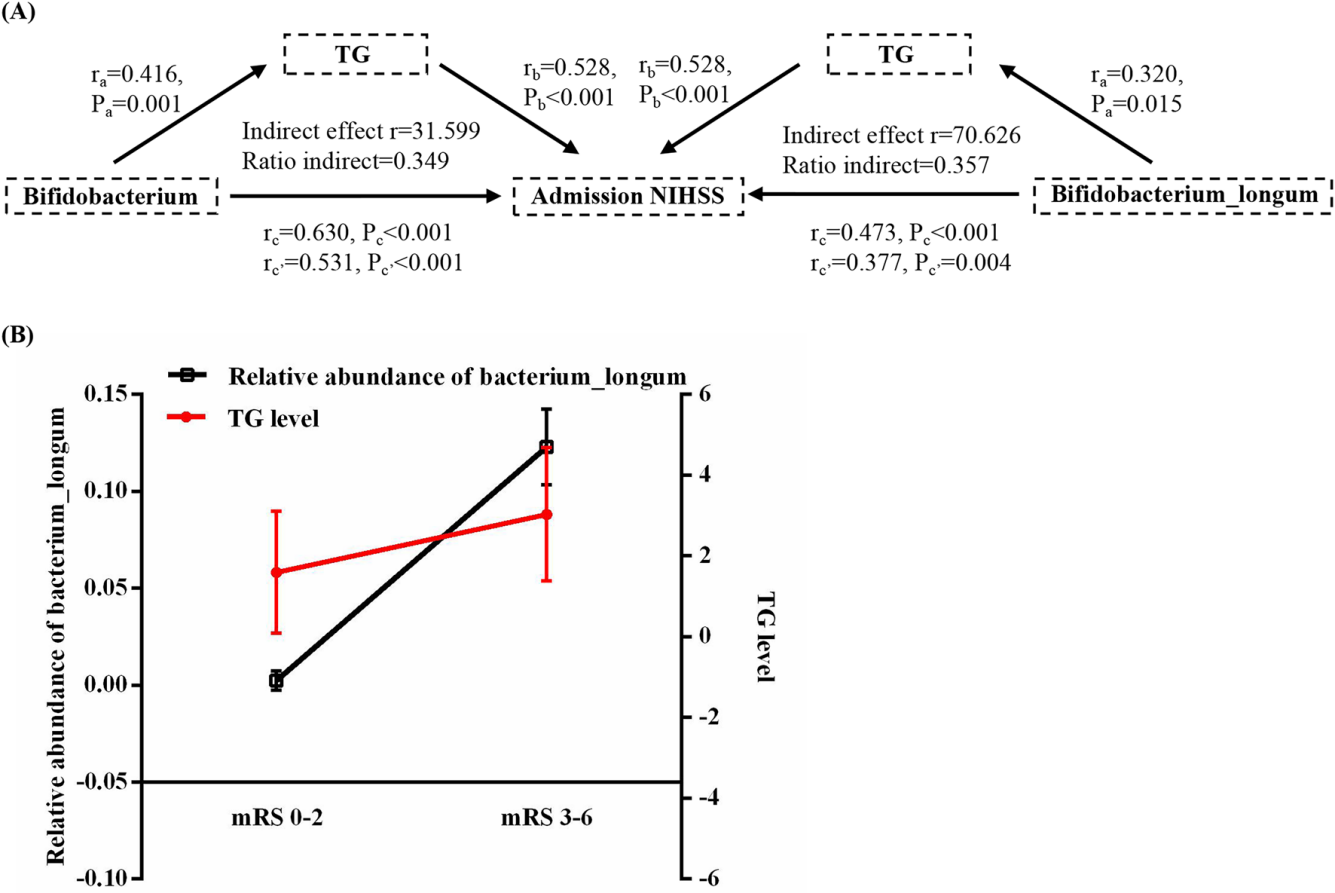
Mediation and interactive effect analyses of gut microbiota in LVO patients. (**A**) The mediation effects of serum TG levels on the relative abundance of genera *Bifidobacterium* and species *Bifidobacterium_longum* and admission NIHSS scores. (**B**) Assessment of interactive effect between serum TG levels and relative abundance of species *Bifidobacterium_longum* on discharge mRS score in LVO patients using the line regression analysis. Covariates were age, sex, BMI, and complications. LVO, large vessel occlusion, NIHSS, National Institutes of Health Stroke Scale; mRS, modified Rankin Scale; TG, triglyceride; BMI, body mass index.

Further mediation analyses in LVO patients depicted that environmental factor significantly regulated the relative abundance of gut microbiota on the severity and outcome of LVO stroke. Those indices that had statistically significance in previous correlation analyses were included in the present mediation analysis. Age, sex, BMI, and complications were used as covariants. The serum TG levels could affect the relative abundance of genera *Bifidobacterium* and species *Bifidobacterium_longum* on the normalized admission NIHSS scores (Fig. 5A).

#### Interaction effect analysis

Additionally, line regression analyses further indicated that higher serum TG levels and higher relative abundance of species *Bifidobacterium_longum* were significantly associated with the worse discharge mRS scores, which suggested that the interactive effects of serum and relative abundance of species *Bifidobacterium_longum* on the discharge mRS scores may occur in LVO patients. Age, sex, BMI, and complications were used as covariants. In order to depict the specific interactive pattern, we divided subjects into a “mRS 0–2” group (good functional outcome group) and a “mRS 3–6” group (poor functional outcome group). LVO patients with higher discharge mRS score showed higher serum TG levels and relative abundance of species *Bifidobacterium_longum* (Fig. 5B).

## Discussion

The main findings of the present study included: (1) the gut microbiota showed altered diversities among these three groups, indicating a higher richness of gut microbiota in the LVO group; (2) the genera *Bifidobacterium*, *Butyricimonas*, *Blautia,* and *Dorea* and the species *Bifidobacterium_longum* showed significantly higher specificity in LVO patients as compared to the NCs and CSVD patients; (3) in LVO patients, the genera *Bifidobacterium* and *Blautia* and species *Bifidobacterium_longum* were significantly associated with the severity of LVO stroke as well as lipid and glucose metabolism; (4) serum TG levels, as an important mediator, could affect the association of the relative abundance of genus *Bifidobacterium* and species *Bifidobacterium_longum* with the NIHSS scores at admission in LVO patients; (5) The relative abundance of genera *Bifidobacterium* and *Blautia* and species *Bifidobacterium_longum* were associated with the functional outcome at discharge in LVO patients. Therefore, the five intestinal microbes were identified in LVO stroke with high specificity, which could reflect the LVO stroke symptoms and function outcome.

To the best of our knowledge, this is the first study, investigating the differences in gut microbiota among NC, CSVD, and LVO stroke. The present study found that the relative abundances of genera *Bifidobacterium* and *Butyricimonas* and species *Bifidobacterium_longum* were significantly higher, while those of genera *Blautia* and *Dorea* were significantly lower in LVO patients than the NC and CSVD groups, suggesting specific changes in the composition and expression of the gut microbiota in LVO stroke. Compared to the single comparison group of NC, a CSVD group was first added to analyze the difference in gut microbiota between the LVO and CSVD groups. CSVD represents the vasculopathy of the small arteries, arterioles, venules, and capillaries of the brain^17^. CSVD is closely correlated with an increased risk of stroke in patients with intracranial arterial stenosis^18^, and high CSVD burden is related to the fast infarct growth rate and poor cerebral collateral circulation in LVO patients^19,20^. This suggests that CSVD is strongly associated with the clinical outcome of LVO stroke. Furthermore, large artery atherosclerosis stroke can affect downstream arterioles and brain tissue hypoperfusion, causing the occurrence of CSVD^21^. Ischemia hypoxia in LVO may induce cellular energy imbalance, oxidative stress, inflammatory response, and other mechanisms to lead to endothelial dysfunction, blood-cerebrospinal fluid barrier injury, and cell apoptosis, which are the primary pathogeny of CSVD^22^. Previous study has also found that the CSVD is more prevalent in the large artery atherosclerosis stroke subtype of LVO stroke^23^. Based on the comparison of three groups, the current study showed that these gut microbiotas had the specific expression in LVO stroke patients, which suggested that the influence of LVO rather than CSVD leaded to these changes in gut microbiotas.

A previous study indicated that the patients with cerebral infarction exhibited a higher abundance of *Bifidobacterium* than the healthy volunteers^24^, which was similar to our findings. However, the findings in the present study were inconsistent with another previous study^25^, which reported a reduced relative abundance of genus *Bifidobacterium* in stroke patients as compared to normal subjects. Due to the modulating effects of oral aspirin and atorvastatin on the human gut microbiota, especially genus *Bifidobacterium* and species *Bifidobacterium_longum*^26^, the collections of fecal samples in the current study were scheduled into 24 h after a thrombolytic therapy with alteplase to reduce the effects of following medication, which represented the gut microbial status closest to the stroke onset. The results showed that the increased relative abundance of genera *Bifidobacterium* and species *Bifidobacterium_longum* were positively correlated with the severity of LVO stroke and negatively correlated with the therapeutic effect of stroke in the LVO group. LVO patients with poor functional outcome exhibited an increased expression of genera *Bifidobacterium* and species *Bifidobacterium_longum* at admission. These indicated that LVO patients with higher relative abundances of genus *Bifidobacterium* and species *Bifidobacterium_longum* at admission might exhibit more severe stroke symptoms and imply a worse efficacy of drug treatment. Furthermore, the present study found significant correlations between the relative abundances of genus *Bifidobacterium* and species *Bifidobacterium_longum* and the serum TG and HCY levels. This suggested that these microbes might be associated with lipid and homocysteine metabolisms in the early stage of LVO stroke^24,27–29^. Meanwhile, in our analyses, the serum TG levels of LVO patients could mediate the effects of species *Bifidobacterium_longum* on the ischemic stroke severity and functional outcome at discharge, which also provided a new insight on high TG as the risk factor of stroke^30,31^. Additionally, ingestion of *Bifidobacterium* was thought to be beneficial for the middle cerebral artery occlusion-induced neurological dysfunctions^32^. The current study showed that the early abundance of *Bifidobacterium* in LVO patients might play a protective role to compete stroke risk factors (hyperlipidemia and hyperhomocysteinemia) and ischemic brain. This protective benefit from *Bifidobacterium* might be exhausted prematurely during treatment if probiotics/prebiotics are not given as dietary supplements, leading to a poor functional outcome.

In the present study, the decrease in the relative abundance of genus *Blautia* was negatively correlated with NIHSS scores at admission and discharge of LVO patients, and LVO patients with good functional outcome exhibited an increased expression of *Blautia* at admission, suggesting that genus *Blautia* abundance was associated with short-term neurological outcomes in stroke. Likewise, Chang et al*.* indicated that acute ischemic stroke patients with good outcomes exhibited an increased abundance of *Blautia*^33^. Furthermore, the current study observed negative correlations between the relative abundance of genera *Blautia* and TG, FBG, and HbAlc levels in the LVO patients, which suggested that *Blautia* might be involved in the glucose and lipid homeostasis in stroke. Previous studies also indicated that beneficial *Blautia* could alleviate hyperglycemia and hyperlipidemia in type 2 diabetes^34,35^. Hence, a supplement of *Blautia* might be beneficial for the treatment of stroke and the improvement of glucose and lipid metabolisms.

The current study showed an increase in the abundance of genus *Butyricimonas* and a decrease in that of genus *Dorea* in LVO patients, which exhibited high specificity for LVO stroke. The patients with middle cerebral artery occlusion-induced ischemic stroke showed an increased abundance of *Butyricimonas*, a pathogenic bacterium, which was significantly correlated with the infarct size and plasma levels of inflammatory markers^36^. Moreover, the abundance of *Butyricimonas* was associated with early neurological deficits and could predict recanalization therapy outcomes in ischemic stroke^37^. In addition, a recent study indicated that *Dorea* could mediate anti-inflammatory metabolites to affect the functional outcomes in ischemic stroke and may be used as a potential target in the clinical therapy of stroke^38^. However, more details on the association between *Butyricimonas*/*Dorea* and stroke are lacking.

There were certain limitations to the current study. (1) The present findings just supported these identified intestinal microbes as a consequence of LVO stroke, and it was unclear whether their changes were the key cause of inducing LVO stroke. (2) The fecal samples were not collected at the end of treatment from the LVO patients. (3) Independent verifications are necessary to determine the specificity of these five gut microbiota in LVO stroke. In a subsequent study, we will further expand the sample size and perform multi-center verifications to identify the characteristics of gut microbiota for large artery atherosclerosis and cardio-embolism stroke subtypes. Moreover, multiple fecal samples should be collected during treatment to explore the dynamic changes in the abundance of gut microbiota, which might contribute to assessing the potential of gut microbiota as a therapeutic target for ischemic stroke. Furthermore, animal experiments are essential to determine whether these changes in gut microbiota can induce the occurrence of LVO stroke.

In conclusion, the present study demonstrated five gut microbes with high specificity in patients with LVO and found several microbes associated with the severity and functional outcome of LVO stroke as well as lipid, homocysteine, and glucose metabolisms. These findings revealed the characteristics of gut microbes in the early stage of LVO stroke and provided a new insight on the stroke clinical management.

## Materials and methods

### Participants

All the participants or their legal guardians provided written informed consent. The Ethics Committee of the Affiliated Wuxi People’s Hospital of Nanjing Medical University approved the current study (approval number: KY21088). All clinical investigations were conducted in strict adherence to the principles outlined in the Declaration of Helsinki, and all experiments were performed in accordance with relevant guidelines and regulations.

A total of 63 LVO patients were recruited from the stroke center of the Affiliated Wuxi People’s Hospital of Nanjing Medical University, and 36 matching NCs were recruited through community health screening in Wuxi City. Moreover, 64 CSVD patients from the Affiliated Wuxi People’s Hospital of Nanjing Medical University, who had been reported in our previous studies, also were included in the present study. The standardized clinical interview was performed for all the participants, involving demographic inventory as well as physical and mental health examination. The brain magnetic resonance imaging (MRI) along with three-dimensional T1-weighted, T2-weighted, fluid-attenuated inversion recovery, susceptibility-weighted images, diffusion-weighted imaging, and magnetic resonance angiography (MRA), were also conducted for each participant. The dietary structure of each participant is similar.

The inclusion criteria for all the participants were as follows: (1) > 18 years old and (2) no contraindication in MRI scan. The LVO patients showed first-ever stroke symptoms, and the time of stroke was less than 4.5 h. All LVO patients were anterior circulation single LVO on computed tomography angiography or MRA. The Pullicino formula was used to calculate the BIV of LVO patients based on the cranial MRI scan. Furthermore, the CSVD patients had periventricular and deep white matter hyperintensity, lacunar infarcts, and cerebral microbleeds but not any new brain infarcts. All the NCs did not have any stroke, which was reflected by consistent imaging results. Participants in the NC group were randomly recruited during the same period. The NC group matched with the LVO and CSVD groups for age, sex, BMI, and complications. Meanwhile, all the participants had no habit of taking antiplatelet drugs or statins prior to enrollment.

The exclusion criteria for each participant were as follows: (1) hemorrhagic disorder or bleeding tendency (e.g., cerebral hemorrhage and gastrointestinal hemorrhage); (2) any severe psychiatric disorders (e.g., schizophrenia and major depressive disorder); (3) any infectious disease (e.g., pulmonary infection); (4) brain trauma or other neurologic diseases (e.g., Parkinson’s disease and Alzheimer’s disease); (5) any significant medical problems (e.g., tumor, significantly impaired liver or kidney functions, digestive system disease, and metabolic disease); (6) record of antibiotic use in the last six months or during treatment; and (7) usage of prebiotics or probiotics within one month before admission or during treatment.

At 24 h after a thrombolytic therapy with alteplase, the LVO patients were arranged for further drug therapies. According to “Chinese Stroke Association Guidelines for Clinical Management of Cerebrovascular Disorders (2019)”^39^, all the ischemic stroke patients with LVO received dual antiplatelet aggregation therapy, lipid-lowering treatment, and improving cerebral microcirculation. The duration of treatment was 8 ± 1 days for each LVO patient. Only those LVO patients, who met the current treatment protocol, were included in this study. Meanwhile, each LVO patient obtained a standard Mediterranean diet during their duration of hospital stay without the use of a nasogastric tube. Notably, the present study was not a clinical trial but an observational study.

Furthermore, NIHSS^40^ scale was used to evaluate the symptoms of stroke, and the mRS^41^ scale was used as a measure of post-stroke physical disability. These two assessments were conducted at the time of admission and discharge of the patients.

### Collection of fecal samples and 16S rRNA gene sequencing

After thrombolytic therapy (within 24 h), the fecal sample was collected after LVO participant’s defecation using stool collection tubes with stool DNA stabilizer (Genstone Biotech, Beijing, China), and then stored at − 80 °C. Furthermore, fecal samples were collected at 7:00–8:00 a.m. for other participants.

FastDNA Spin Kit For Soil (MP Biomedicals, Santa Ana, CA) was used for the DNA extractions of fecal samples. Subsequently, the compositional analysis of gut microbiota were conducted by Genesky Biotechnologies Inc. (Shanghai, China). Details of sequencing and data analysis could be found in Supplementary Materials and previous studies^42,43^.

### Collection of serum samples and detection of blood indexes

For LVO patients, peripheral venous blood was collected using a vacutainer tube (without anticoagulant) and an EDTA-coated tube at 6:00–7:00 a.m. on the first day after admission. The serum samples were further obtained by centrifugation at 3500 rpm at 4 °C for 10 min. All blood samples were measured immediately after collection. Due to the main goal of the present study is to investigate specific gut microbiota of LVO and potential association of gut microbiota with the pathologic change in LVO stroke, blood samples were collected only in the LVO group.

The total cholesterol, TG, low-density lipoprotein cholesterol, high-density lipoprotein cholesterol, FBG and uric acid were detected in serum using a fully automatic biochemical analyzer (UniCel DxC 600 Synchron, Beckman Coulter, California, United States). Hemoglobin A1c (HbA1c) level was assayed in whole-blood samples and HCY level was assayed in serum samples using the high-performance liquid chromatography (Agilent 1200 device, Agilent Technologies, Waldbronn, Germany).

### Statistical analyses

The data analyses were conducted using SPSS version 23.0 (SPSS Inc. Chicago, IL, USA) and R software package (version 4.2.1).

Alpha and beta diversity analyses were performed for the diversity analysis of gut microbiota. In alpha-diversity analysis, Observed species, Chao1, and ACE indices were used for assessing the community richness, and Shannon, Simpson, and Coverage indices were used for assessing the community diversity. Beta diversity was used to analyze differences in the composition of gut microbiota using PCoA and PLS-DA. LEfSe was performed to identify the markers to interpret the difference among groups where the threshold score of LDA was 2. The functions of species in the gut microbiota were predicted using PICRUSt2 analysis tool and Kyoto Encyclopedia of Genes and Genomes database (https://www.genome.jp/kegg/pathway.html).

The continuous variables were shown as mean ± standard deviation and were analyzed using the Kruskal–Wallis H test for non-normal distribution, or the One-way ANOVA test for normal distribution. The categorical variables were analyzed using the Chi-square test. Partial correlation analysis was used to determine the correlation between the taxonomies of gut microbiota and clinical data in LVO patients, with adjusting age, sex, BMI, and complications. Furthermore, the mediation analysis in LVO patients could determine whether environmental factors mediated the association between the relative abundance of gut microbiota on the severity of stroke based on a standard three-variable mediation model^44,45^. Line regression was used to evaluate the interaction between two variables on the functional outcome^46,47^. Changed value = admission score − discharge score. The statistically significant differences were considered as p-value < 0.05.

### Institutional review board statement

The Ethics Committee of the Affiliated Wuxi People’s Hospital of Nanjing Medical University approved the current study (approval number: KY21088).

### Informed consent

A signed written informed consent form and acceptance to participate in the study were received from all participants in the study.

### Supplementary Information

Supplementary Information.

## Data Availability

The data supporting this study’s findings are available on request from the corresponding author.
