# Supplementary material for: Identifying gut microbiota with high specificity for ischemic stroke with large vessel occlusion
Source: Sci Rep. 2024 Jun 18;14:14086. doi: 10.1038/s41598-024-64819-6 (PMC11189444; doi:10.1038/s41598-024-64819-6)
Supplement: Supplementary file 1 — Supplementary Information. [file 41598_2024_64819_MOESM1_ESM.docx]

#### 16S rRNA gene sequencing

The DNA from fecal samples (500 mg for each sample) was extracted using FastDNA Spin Kit For Soil (MP Biomedicals, Santa Ana, CA) following the manufacturer’s guidelines. The integrity and quality of genomic DNA were determined by agarose gel electrophoresis, and NanoDrop 2000 (10x Genomics, USA) and Invitrogen Qubit 3.0 Spectrophotometer (Thermo Fisher Scientific, USA), respectively. The V3-V4 hyper-variable regions of the 16S rRNA gene were amplified with the primers 341F (5’-CCTACGGGNGGCWGCAG-3’) and 805R (5’-GACTACHVGGGTATCTAATCC-3’) using a high-fidelity polymerase chain reaction (PCR). The DNA mix of the standard bacterial genomes as a positive control was used for 16S rRNA gene sequencing. The uniqueness and specificity of the above-mentioned amplified products were examined with agarose gel electrophoresis. The PCR products were purified using Agencourt AMPure XPPCR Purification Beads (Beckman Coulter, USA). The concentration of the index labeled sample gene library was diluted 5-10 times according to the preliminary quantitative result shown by agarose gel using Qubit. The length of the inserted fragment was measured using Agilent 2100 Bioanalyzer (Agilent Technologies, USA). Illumina NovaSeq 6000 (Illumina, USA) sequencing platform was utilized to sequence the library.

#### Data processing and analysis of sequencing

The raw sequence reads were analyzed with Quantitative Insights Into Microbial Ecology (QIIME 2). The Cutadapt plugin was utilized to tailor the adaptor and primer sequences. The quality control and identification of amplicon sequence variants (ASVs) were conducted using Divisive Amplicon Denoising Algoruthm 2 (DADA2). The taxonomic assignments of ASV representative sequences were conducted according to a confidence threshold of 0.8 using a pre-trained Naive Bayes classifier, which was trained on the Ribosomal Database Project (RDP) classifier (version 11.5).

**Supplementary Table 1. Clinical characteristic of 63 LVO participants in the present study.**

|  | Cardioembolism | Large-artery atherosclerosis | P-value |
| --- | --- | --- | --- |
| Number | 13 | 50 | - |
| Age (years) | 69.85 ± 10.25 | 66.58 ± 11.56 | 0.358^*^ |
| Sex (Male, %) | 10 (76.92%) | 33 (66.00%) | 0.524^#^ |
| BMI | 24.53 ± 2.27 | 24.53 ± 2.44 | 0.994^*^ |
| Complications (N, %) |  |  |  |
| Hypertension | 10 (76.92%) | 33 (66.00%) | 0.524^#^ |
| Diabetes | 6 (46.15%) | 17 (34.00%) | 0.417^#^ |
| Coronary heart disease | 3 (23.08%) | 0 (0.00%) | 0.001^#^ |
| Admission NIHSS scores | 8.54 ± 5.92 | 7.52 ± 6.99 | 0.404^&^ |
| Discharge NIHSS scores | 4.62 ± 4.99 | 4.86 ± 6.59 | 0.569^&^ |
| Admission mRS scores | 3.23 ± 1.24 | 3.16 ± 1.36 | 0.944^&^ |
| Discharge mRS scores | 2.00 ± 1.63 | 1.58 ± 1.91 | 0.298^&^ |
| BIV (cm^3^) | 16.58 ± 26.99 | 21.52 ± 59.21 | 0.255^&^ |
| Stroke blood risk indices |  |  |  |
| TC (mmol/L) | 4.85 ± 1.14 | 4.60 ± 1.36 | 0.542^*^ |
| TG (mmol/L) | 1.69 ± 1.30 | 2.18 ± 1.78 | 0.197^&^ |
| LDL-C (mmol/L) | 2.44 ± 0.85 | 2.64 ± 0.91 | 0.471^*^ |
| HDL-C (mmol/L) | 1.14 ± 0.36 | 1.17 ± 0.56 | 0.993^&^ |
| FBG (mmol/L) | 6.69 ± 1.70 | 6.81 ± 2.63 | 0.869^*^ |
| HbAlc (%) | 6.84 ± 1.29 | 6.78 ± 1.67 | 0.524^&^ |
| HCY (μmol/L) | 16.62 ± 7.04 | 14.78 ± 5.03 | 0.282^*^ |
| UA (μmol/L) | 259.96 ± 145.12 | 296.61 ± 98.84 | 0.287^*^ |


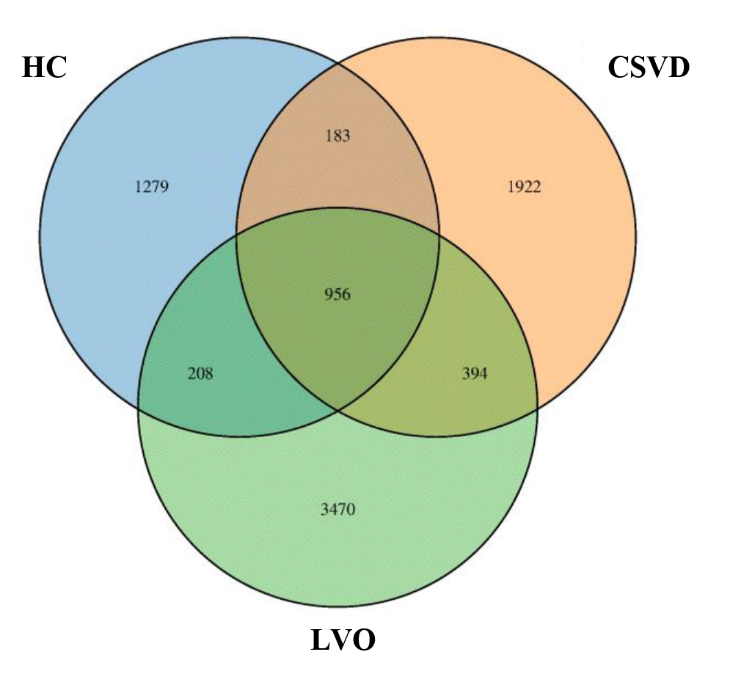


**Supplementary Figure S1. Venn of the distribution of gut microbiota in the three groups.**


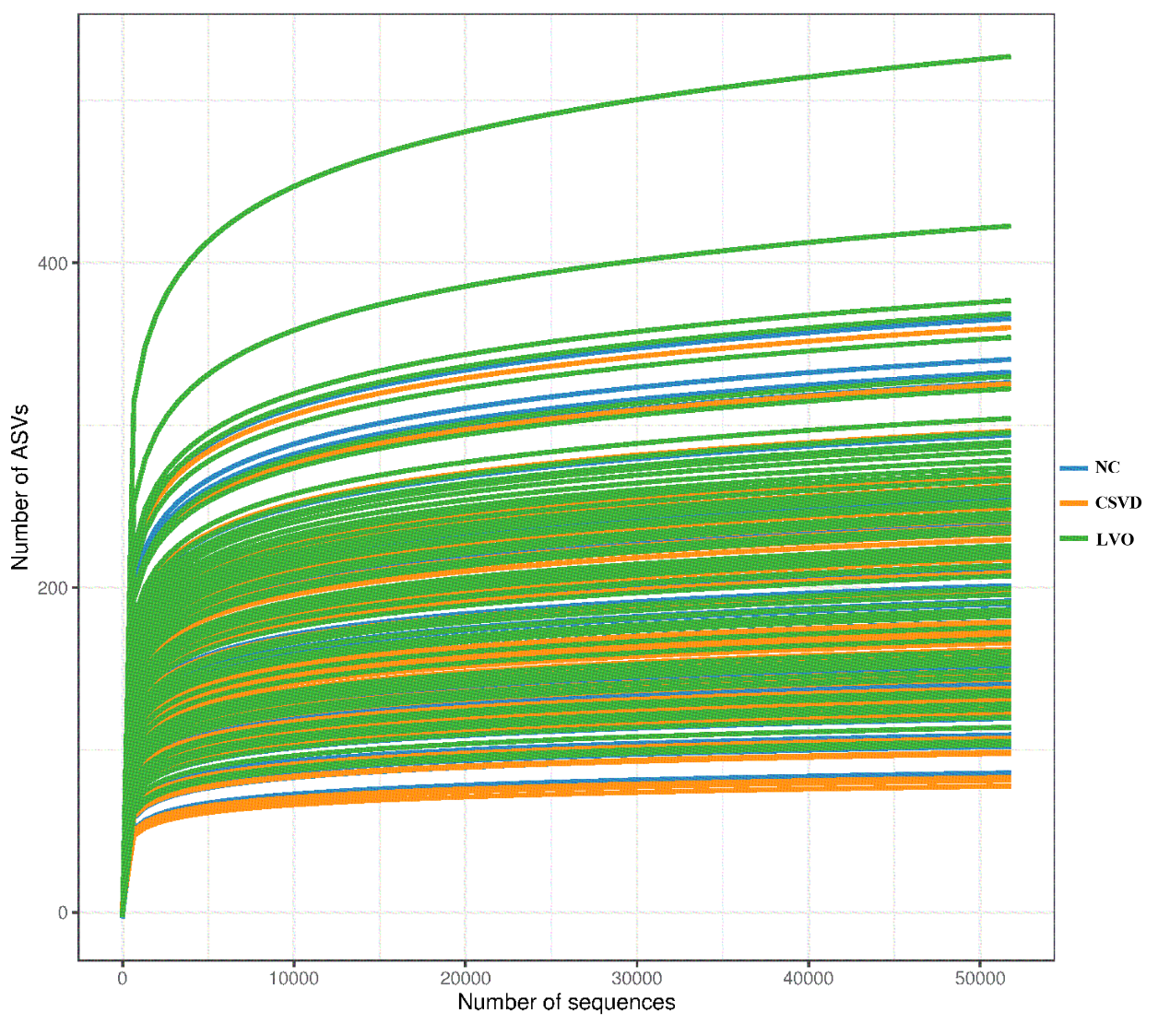


**Supplementary Figure S2. All the samples’ curves in the rarefaction curves.**


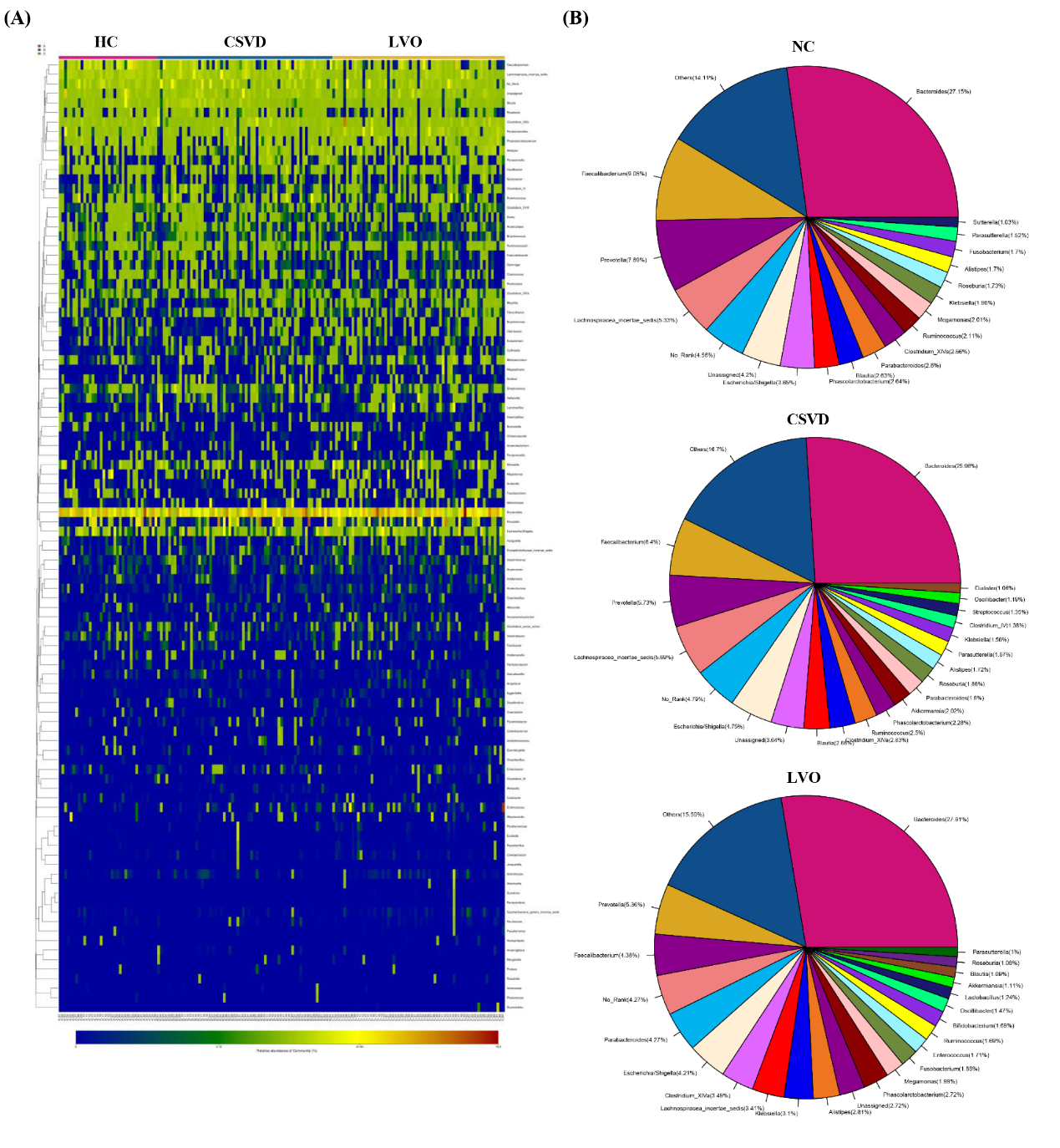


**Supplementary Figure S3. Relative abundances at genus level.**

(A) Heat-map analysis at genus. Abscissa is the sample and ordinate is the taxa at genus level. The colors in heat-map represent the genus abundance, and the gradual change of color from blue to red indicates that the genus abundance changed from small to large. (B) Bar plots of the relative abundances of three groups at genus level.


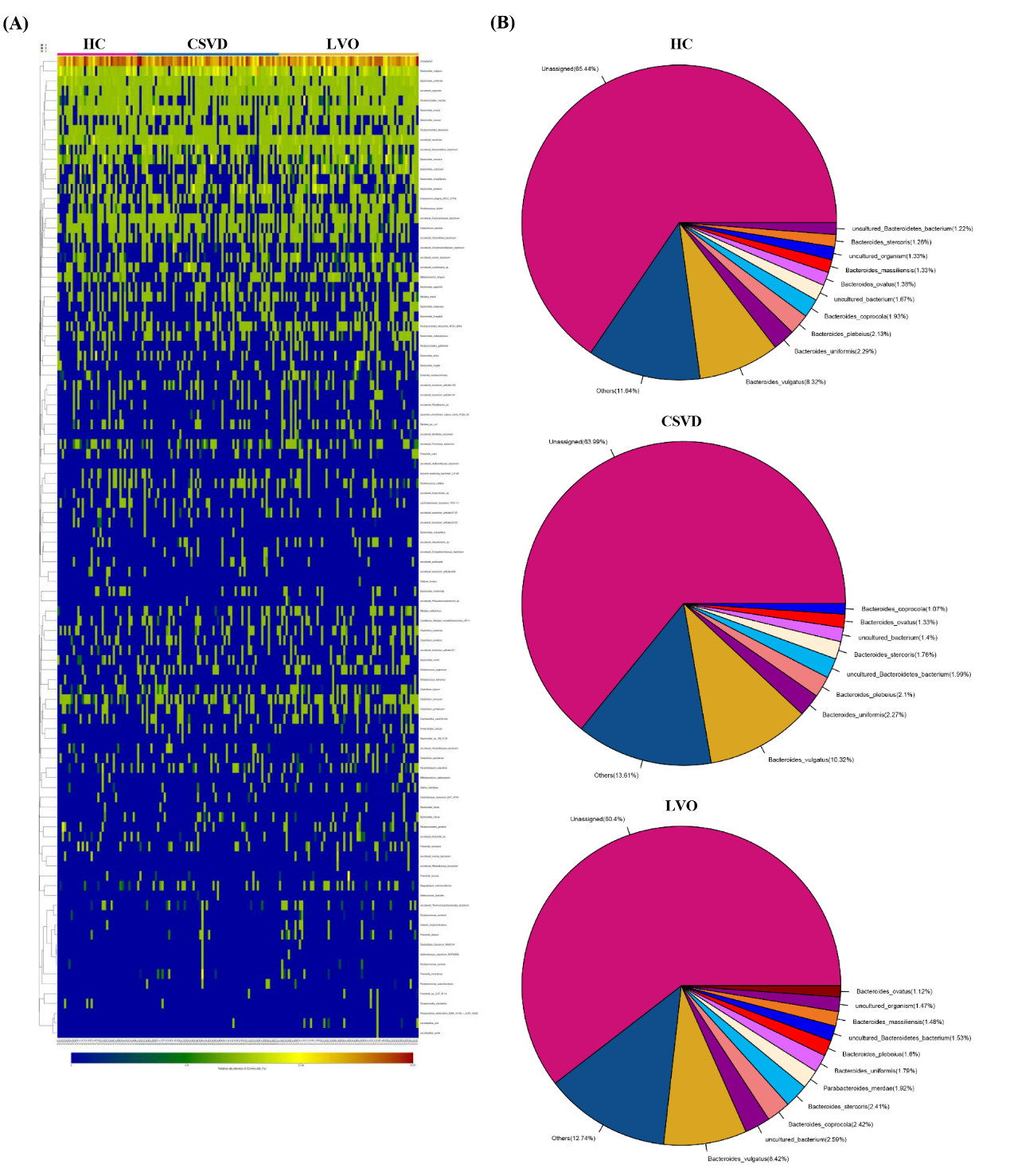


**Supplementary Figure S4. Relative abundances at species level.**

(A) Heat-map analysis at species. Abscissa is the sample and ordinate is the taxa at species level. The colors in heat-map represent the species abundance, and the gradual change of color from blue to red indicates that the species abundance changed from small to large. (B) Bar plots of the relative abundances of three groups at species level.


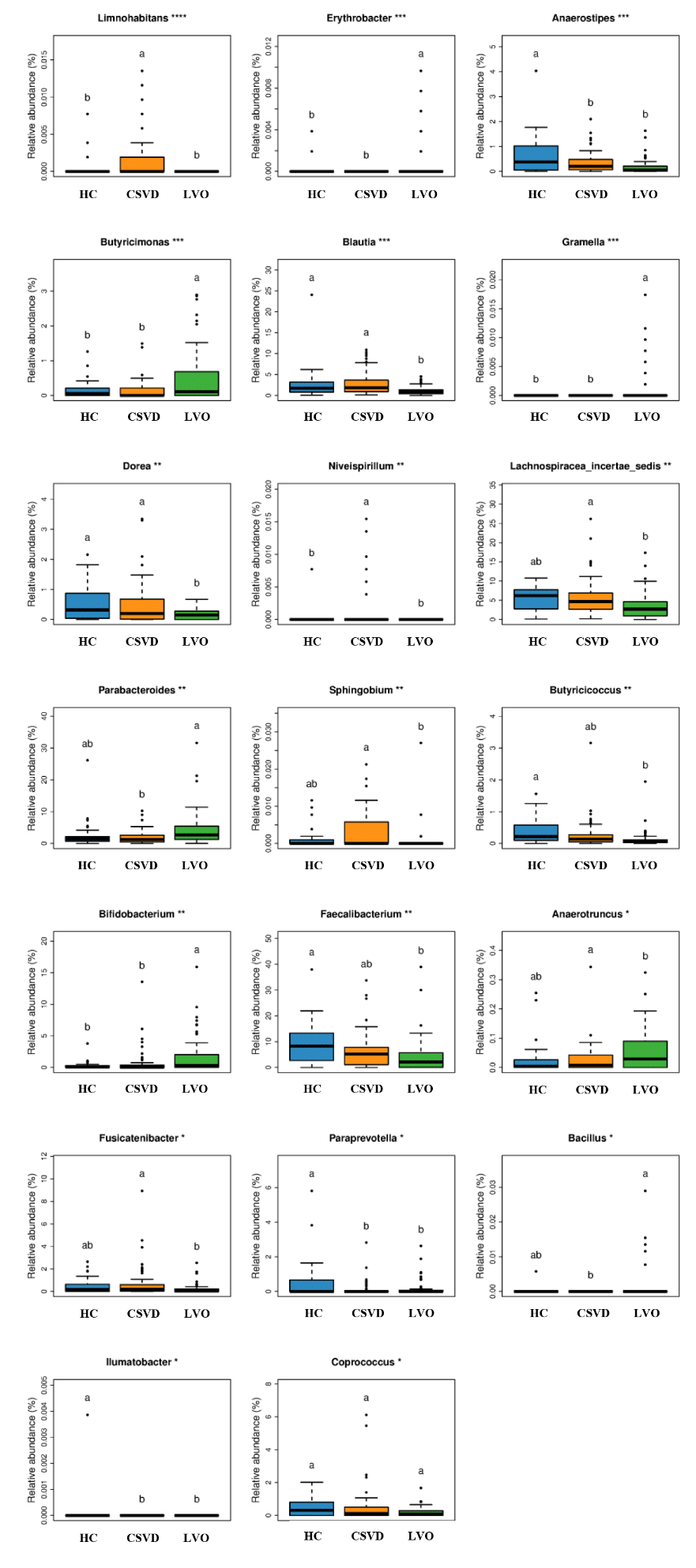


**Supplementary Figure S5. Taxa with significant differences among the NC, CSVD and LVO groups at phylum level.**


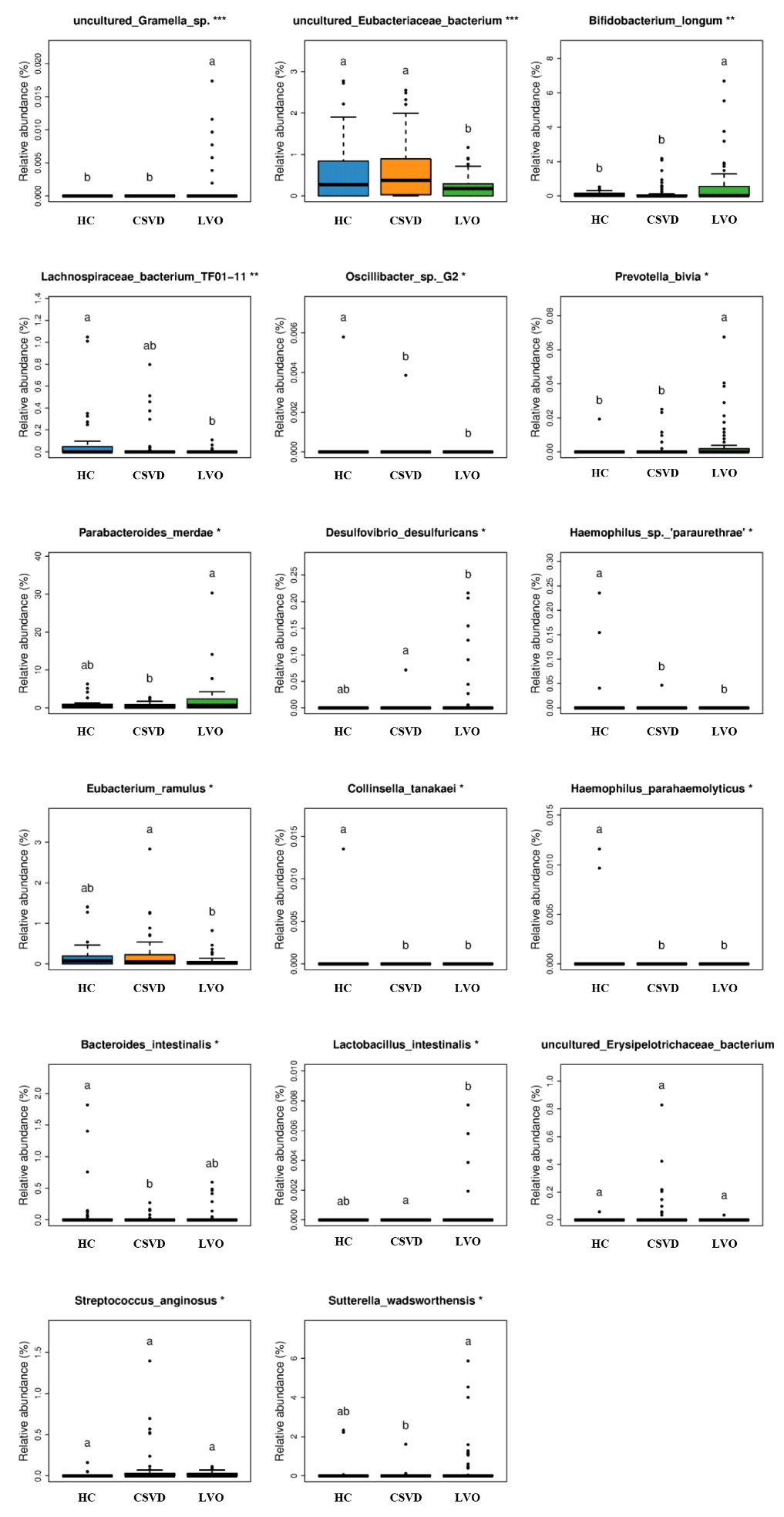


**Supplementary Figure S6. Taxa with significant differences among the NC, CSVD and LVO groups at species level.**


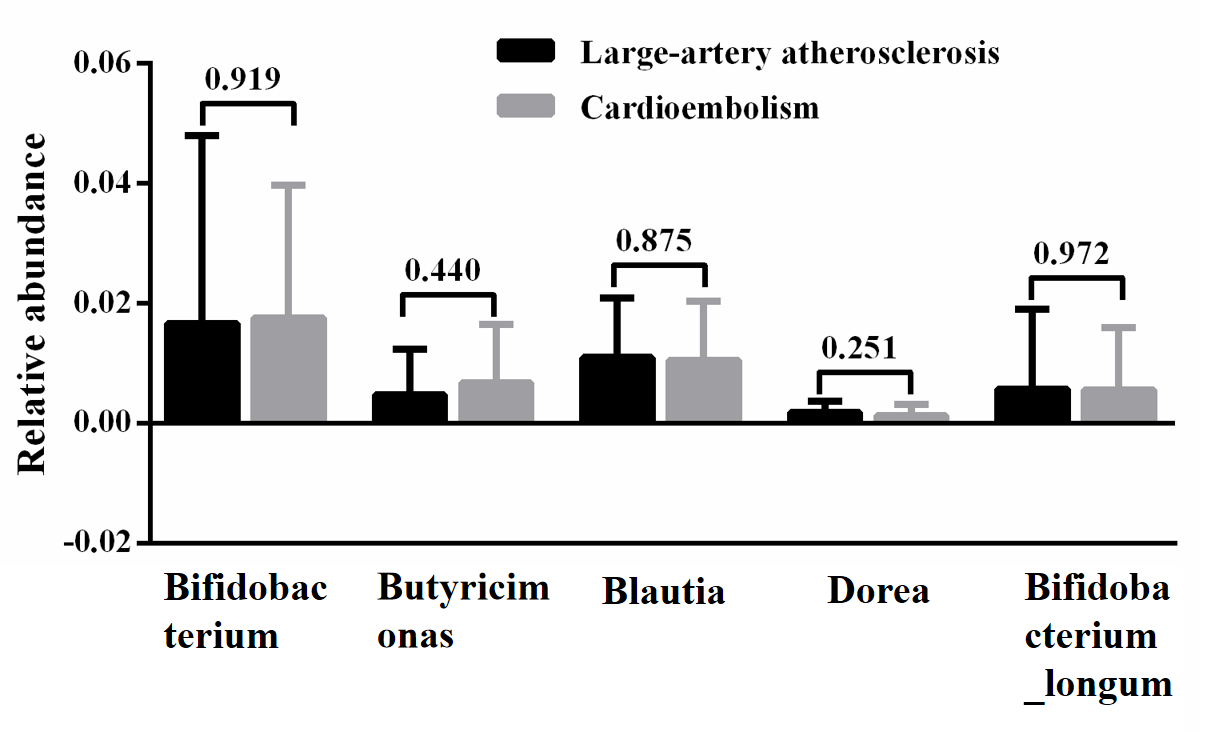


**Supplementary Figure S7. Different abundances of four genera and one species between large-artery atherosclerosis and cardioembolism subgroups in LVO patients.**


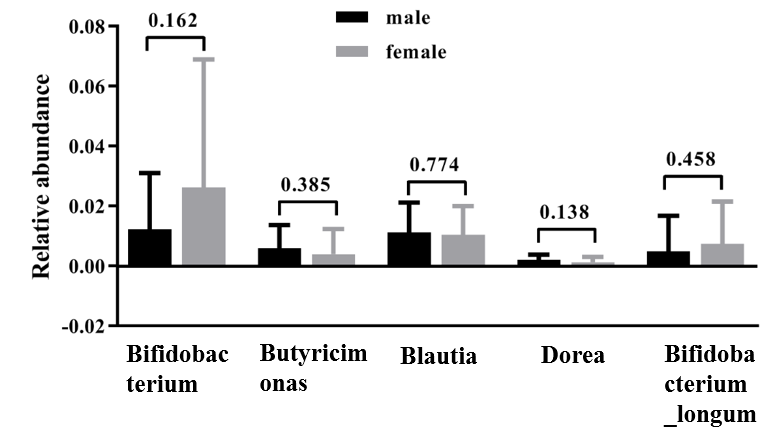


**Supplementary Figure S8. Different abundances of four genera and one species between male and female subgroups in LVO patients.**


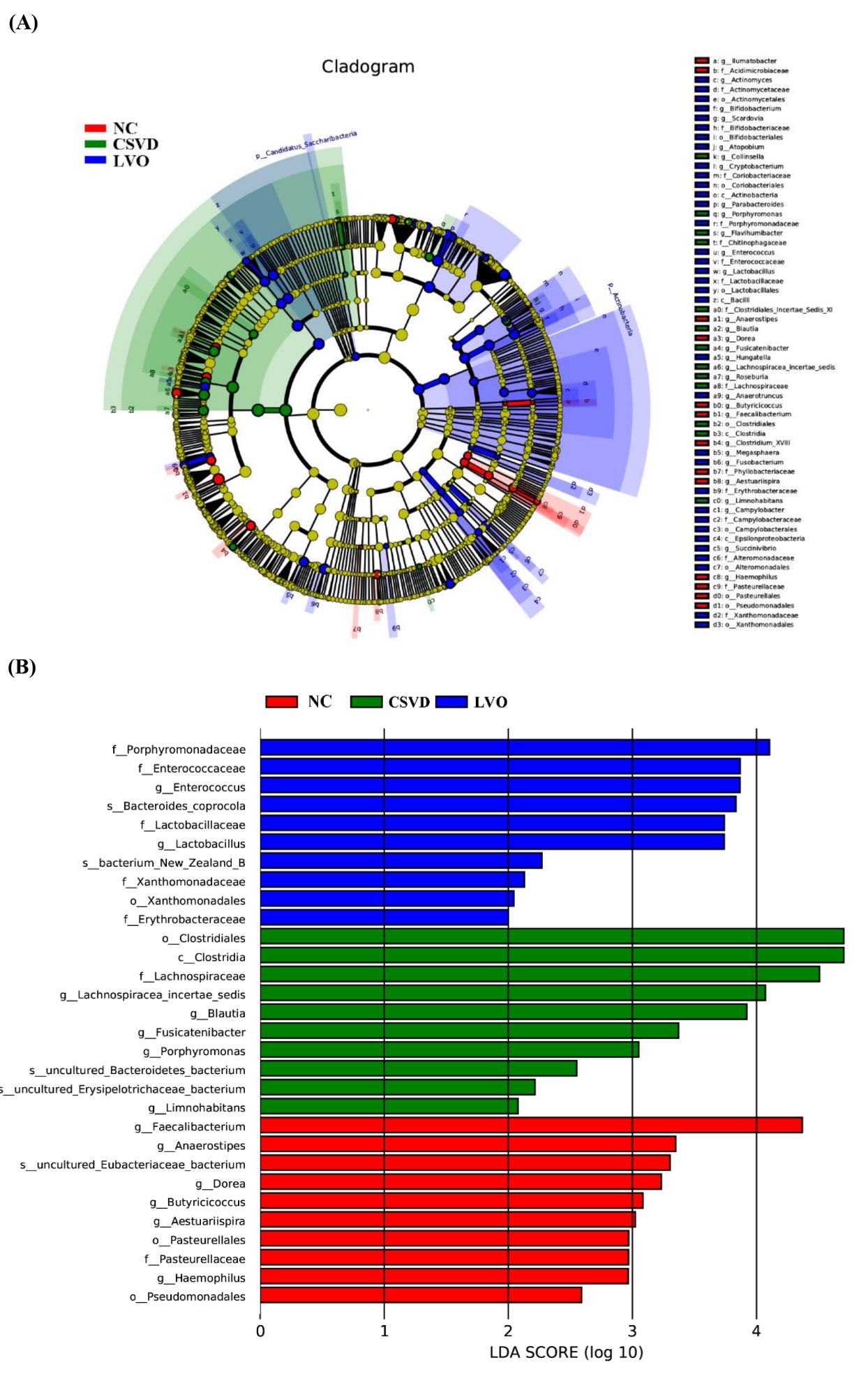


**Supplementary Figure S9. Taxa with different abundances according to LEfSe analysis in the NC, CSVD and LVO groups.**

(A) Cladogram generated by LEfSe. Statistically significant differences (LDA scores > 2) in relative abundance of top 50 taxa with the minimum P-value among NC, CSVD and LVO participants, P < 0.05. Red, green and blue nodes indicate enriched taxa in the NC, CSVD and LVO groups, respectively. The diameter of each node shows the relative abundance of each taxon and is proportional to the observed effect size. (B) Histogram of the LDA scores (>2) computed for the top 10 taxa with minimum P-value. Red, green and blue bars indicate taxa were enrichment in the NC, CSVD and LVO groups, respectively.
